# Inhibition of STAT3 signaling as critical molecular event in resveratrol-suppressed ovarian cancer cells

**DOI:** 10.1186/s13048-015-0152-4

**Published:** 2015-04-22

**Authors:** Li-Xia Zhong, Hong Li, Mo-Li Wu, Xiao-Yu Liu, Ming-Jun Zhong, Xiao-Yan Chen, Jia Liu, Yang Zhang

**Affiliations:** Department of Clinical Oncology, Second Affiliated Hospital of Dalian Medical University, Dalian, 110042 China; Liaoning Laboratory of Cancer Genetics and Epigenetics and Department of Cell Biology, Dalian Medical University, Dalian, 116044 China

**Keywords:** Ovarian cancer, Resveratrol, Signal transduction pathway, STAT3, Selective inhibitor, Gene expression

## Abstract

**Background:**

Resveratrol exerts inhibitory effects on ovarian cancer cells, while its underlying mechanism and critical molecular target(s) have been lesser known. Activations of Wnt, Notch and STAT3 signaling are frequent in ovarian cancers/OCs and supposed to be important for OC formation and progression, while the impacts of resveratrol on these signaling pathways in OC cells remain obscure.

**Methods:**

In this study, two human ovarian cancer cell lines, OVCAR-3 and CAOV-3, were treated by 120 μM resveratrol and their responses to the treatment and the statuses of Wnt, Notch and STAT3 signaling in them were analyzed by multiple experimental approaches. Selective inhibitors of Wnt, Notch or STAT3 signaling were employed to treat OVCAR-3 and CAOV-3 cells to elucidate the significance of individual signaling pathways for ovarian cancers.

**Results:**

The results demonstrated distinct inhibitory effects of resveratrol on human ovarian cancer cells in terms of remarkable G1 phase accumulation, increased apoptosis fraction and concurrent suppression of Wnt, Notch and STAT3 signaling as well as their downstream cancer-related gene expression. Treatments with Wnt, Notch or STAT3 selective inhibitor revealed that only AG490, a JAK-specific inhibitor, inhibits OVCAR-3 and CAOV-3 cells in the extent as similar as that of resveratrol.

**Conclusion:**

Our results suggest the significance of STAT3 activation in the maintenance and survival of ovarian cancer cells. The activated STAT3 signaling is the critical molecular target of resveratrol. Resveratrol would be a promising candidate in the management of ovarian cancers, especially the ones with resistance to conventional therapeutic agents.

## Introduction

Ovarian cancer (OC) is one of the commonest female malignancies and accounts for the leading death rates among the gynecologic cancers [1,2]. The main reasons of the poor prognosis of OCs are the delayed diagnosis due to the very subtle symptoms at the early stage of ovarian carcinogenesis [3] and the easiness of spreading through blood dissemination [4] and peritoneal transplantation [5,6]. Surgical treatment is the first choice to remove ovarian cancers if the tumours are well-differentiated, in relative small sizes and/or confined to the ovary [7,8]. However, the patients with advanced OCs have to be operated for debulking the disease and then treated by standard chemotherapy such as a dose-dense paclitaxel and carboplatin regimen [9,10]. Although the therapeutic outcome has been improved by more accurate staging of the disease and more aggressive surgical excision of tumor spots in the abdomen, the overall survival rates remain unoptimistic because of the frequent tumour recurrence and severe toxic effects of the anticancer agents [11-13]. For these reasons, it would be necessary to explore more efficient and lesser toxic agent(s) with clearer molecular targets for better adjuvant management of ovarian cancers.

Resveratrol (3,5,4′-trihydroxy-*trans*-stilbene) has been regarded as a non-toxic polyphenolic compound that can be found in grapes, berries, peanuts and red wine [14]. A body of evidence has demonstrated that resveratrol is able to inhibit the growth of many cancers such as bladder cancer, breast cancer and primary brain tumors [15-17]. Increasing data have shown that resveratrol can exert its biological effects on cancer cells by altering multiple molecular targets [18,19]. For example, it suppresses growth and induces apoptosis of human medulloblastoma cells accompanied with inhibition of STAT3 activation and transcription [18]. More importantly, the anticancer doses (100 μM to 200 μM) of resveratrol have little harmful effect on glial cells and neurons in central nervous system and transitional epithelial cells of the urinary bladder [15,17,19]. The inhibitory effects of resveratrol on ovarian cancer cells have been documented as well [20,21]. Although some studies have shown certain molecular alterations in resveratrol-treated ovarian cancer cells, such as down-regulation of Akt/GSK signaling [22] and VEGF expression [23], the critical event(s) among those alterations remains largely unknown. It is therefore necessary to address this point by comprehensively analyzing the statuses of ovarian cancer-related signaling pathways as well as their downstream genes.

Some signaling transduction pathways are found to be activated in the processes of ovarian carcinogenesis and play favorable roles in cell growth and survival [24-26]. For instance, hyperactive Jaks/STAT3 signaling promote enhanced colony-forming ability, motility and migration of cisplatin-resistant ovarian cancer cells [27]. Similarly, Wnt/beta-catenin pathway also contributes to the proliferation of human ovarian cancer cell [28] and inhibition of Notch signaling, a key pathway for ovarian cancer stem cells, sensitizes tumors to platinum therapy [25]. The data obtained from other cancer systems reveal that resveratrol can inhibit the signaling pathways mediated by STAT3, Wnt and Notch when exerting its cancer suppressive effects [18,29,30]. The current study thus refers to the above findings as a cue and/or a cutting edge to identify the critical molecular event(s) caused by resveratrol in ovarian cancer cells.

## Materials and methods

### Cell culture and treatment

Human ovarian cancer CAOV-3 cells [31] were cultured in Dulbecco’s modified Eagle’s essential medium (DMEM) containing 12% fetal bovine serum (Gibco Life Science, Grand Island, NY, USA) under 37°C and 5% CO_2_ condition and OVCAR-3 cells [32] in Roswell Park Memorial Institute 1640 Medium (RPMI1640) under 37°C and 5% CO_2_ condition. The cells (5 × 10^4^/ml) were plated to culture dishes (NUNC, Denmark) and incubated for 24 h before the experiments. Meanwhile, dozens of cell-bearing coverslips were concurrently prepared using the Nest-Dishes (Nest Biotech. Inc., Wuxi, China; China invention patent No. ZL200610047607.8); they were collected from Nest-Dishes, incubated under different experimental conditions and then harvested for H/E morphological staining, immunocytochemical (ICC) labeling and TUNEL assay. Resveratrol (Res; Sigma Chemical, Inc, St. Louis, MO, USA) was dissolved in dimethylsulfoxide (DMSO; Sigma) and diluted with culture medium to the working concentrations just before use. The cells were treated by 100 μM [16,18,19] or 120 μM Res for 72 hours. The normally cultured cells and the cells treated by 0.2% DMSO were used as normal and background controls, respectively. Cell numbers and viabilities were checked in 24 h intervals. The experimental groups were set in triplicate and the experiments were repeated at least for three times to establish confidential conclusion.

### Evaluation of cell growth

The effects of resveratrol on cell proliferation were determined by 3-[4,5-Dimethylthiazol-2-yl]-2,5-diphenyl-tetrazolium bromide (MTT) assay [17]. The results were shown as percentage of cell viability (OD of the experiment samples/OD of control) or OD values. And the fractions of viable and unviable cells in normally cultured and resveratrol-treated populations were estimated with cell counting apparatus (TC20 Automated Cell Counter, BIO-RAD Inc., Singapore). Terminal deoxynucleotide transferase (TdT)–mediated dUTP-biotin nick-end labeling (TUNEL) assay was employed to detect apoptotic cells according to producer’s instructions (Promega Corporation, USA). Haematoxylin and eosin (H/E) staining was performed on the three groups of OVCAR-3 and CAOV-3 cells to evaluate their morphological features.

### Flow cytometry

The harvested cells of the experimental groups were fixed in 70% ethanol for staining with DNA dye, and then suspended in 0.5 ml to 1 ml of propidium iodide solution containing RNase and incubated at 37°C for 30 minutes. Cell cycle profiles and cell apoptotic fractionations were obtained with a FACSvantage flow cytometer (Becton Dickinson, San Jose, CA, USA) and the data were analyzed with ModFit software (Verity Software House, Inc, Topsham, ME). The analyses were repeated for three times to establish confidential conclusion.

### Immunocytochemical staining

Immunocytochemical staining (ICC) was performed on the cell-bearing coverslips of the three experimental groups by the method described previously [18]. The antibodies against human STAT3, p-STAT3, Notch1, Notch2, HES1, Wnt2, β-catenin, E-cadherin, Bcl-2, c-Myc, survivn were purchased from Santa Cruz Biotechnology, Inc, CA and HES1 was provided by Dr. Tetsuo Sudo as a generous gift [29]. Color reaction was developed using 3, 3′-diaminobenzidine tetrahydrochloride (DAB). According to the labeling intensity, the staining results were evaluated by two independent researchers and scored as negative (−) if no immunolabeling was observed in target cells, weakly positive (+) if the labeling was faint, moderately positive (++), and strongly positive (>++) when the labeling was stronger or distinctly stronger than (++).

### RNA isolation and RT-PCR

Sample RNAs were isolated from the two ovarian cancer cell lines cultured under different conditions for 48 hours using Trizol solution (Life Technologies, Grand Island, NY, USA). By the method described elsewhere [8], reverse transcription (RT) was performed on RNA samples, followed by polymerase chain reaction (PCR) with a pair of primers specific for the cDNA of an individual gene (Table 1). The PCR products were resolved on 1% agarose gel containing ethidium bromide (0.5 μg/ml), visualized and photographed using UVP Biospectrum Imaging System (UVP, Inc, Upland, CA). The β-actin PCR products generated from the same RT solution were cited as quantitative controls.

**Table 1:** **Sequences of RT-PCR Primers and their generated product**

**Parameters**	**Primer sequences**	**Product size (bp)**	**Reference**
Notch1	F:5′ - TGT GAC AGC CAG TGC AAC TC - 3′	577	[29]
	R:5′ - TGG CAC TCT GGA AGC ACT GC - 3′		
Notch2	F:5′-AAT GTC ATG GCC GCT TCA GAG-3′	533	[29]
	R:5′-TCG TGC AAG AGC CAG TTA CCC-3′		
Hes1	F:5′ - CCA GTT TGC TTT CCT CAT TCC - 3′	240	[29]
	R:5′ - TCT TCT CTC CCA GTA TTC AAG TTC C - 3′		
Wnt2	F:5′ - GCC ACA CGC TGC ACC TAA AGC - 3′	379	[30]
	R:5′ - CAA TTA CCC TAA GGG TGG TAG C - 3′		
β-catenin	F:5′-TGA TGG AGT TGG ACA TGG CCA TGG-3′	570	[53]
	R:5′-CAG ACA CCA TCT GAG GAG AAC GCA-3′		
E-cadherin	F:5′-GAC GCG GAC GAT GAT GTG AAC-3′	281	[30]
	R:5′- TTG TAC GTG GTG GGA TTG AAG A-3′		
STAT3	F:5- GGG TGG AGA AGG ACA TCA GCG GTA A-3′	298	[15]
	R:5′- GCC GAC AAT ACT TTC CGA ATG C −3′		
survivin	F:5′-GGC ATG GGT GCC CCG ACG TTG-3′	439	[15]
	R:5′-CAG AGG CCT CAA TCC ATG GCA-3′		
c-Myc	F:5′-TGG TCT TCC CCT ACC CTC TCA AC −3′	265	[15]
	R:5′-GAT CCA GAC TCT GAC CTT TTG CC −3′		
Bcl-2	F:5′-TTT GAG TTC GGT GGG GTC AT −3′	275	[54]
	R:5′-TGA CTT CAC TTG TGG CCC AG −3′		

### Protein preparation and Western blotting

Total cellular proteins were prepared from the cells under different culture conditions. The sample proteins (50 μg/well) were separated in 10% sodium dodecylsulfate-polyacrylamide gel electrophoresis and transferred to polyvinylidene difluoride membrane (Amersham, Buckinghamshire, UK). The membrane was blocked with 5% skimmed milk in TBS-T (10 mM Tris–HCl, pH8.0, 150 mM NaCl and 0.5% Tween 20) at 4°C, rinsed 10 minutes for three times with TBS-T, followed by 3 h incubation at room temperature with the first antibody in appropriate concentrations (Notch1: 1:800; Notch2: 1:800; Hes1: 1:2500; Wnt2: 1:800; β-catenin: 1:800; E-cadherin: 1:600; STAT3: 1:800; Bcl-2: 1:800; c-Myc: 1:600; survivin: 1:800), and then 1 h incubation with HRP-conjugated anti-mouse or anti-rabbit IgG (Zymed Lab, Inc). The bound antibody was detected using the enhanced chemiluminescence system (Roche GmbH, Mannheim, Germany). After removing the labeling signal by incubation with stripping buffer [8], the membrane was reprobed with other antibodies one by one until all of the parameters were examined.

### Selective inhibition of activated Wnt, Notch and STAT3 signaling

L-685,458 (Calbiochem, San Diego, CA) is a potent and selective γ-secretase inhibitor, which inhibits Notch activation [33]. XAV-939 (Selleck, Houston, Texas, USA) selectively suppresses the transcription of Wnt/β-catenin through inhibiting tankyrase1/2 [34]. AG490 (Sigma, Inc, St. Louis, MO), a JAK-specific inhibitor, can suppress STAT3 signaling by inhibiting Tyr705 phosphorylation of STAT3 protein [19]. To evaluate the importance of the three signalling pathways in the growth and survival of ovarian cancer cells, CAOV-3 and OVCAR-3 cells were treated by 8 μM L-685,458, 10 μM XAV-939 and 80 μM AG490, respectively [35]. The treatments lasted for 72 hours and the cells were observed in 12 hour intervals. The cell bearing coverslips prepared from each of the treatments were subjected to further analyses. The experiments were repeated for 3 times.

### Statistical analysis

MTT data and cell counting were evaluated with the independent-samples t-test and one-way ANOVA. Statistical significance was defined as *P* < 0.05.

## Results

### Resveratrol caused growth arrest and apoptosis

**Figure 1:**
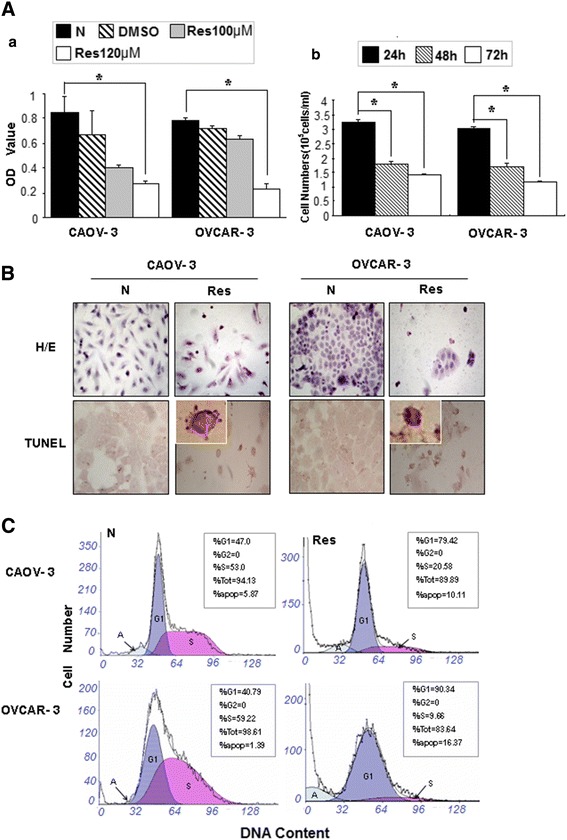
Evaluation of cellular responses of human ovarian cancer CAOV-3 and OVCAR-3 cells to 120 μM resveratrol treatment. **A.** (a) MTT cell proliferation assay performed on the two cell lines cultured normally (N) and incubated in 0.02% DMSO containing medium without (DMSO) or with 100 μM and 120 μM resveratrol supplementation (Res) for 48 hours. *, compared with N group, *P* < 0.05, (b) Viable cell counting of 120 μM resveratrol-treated CAOV-3 and OVCAR-3 cells at 24 h, 48 h and 72 h points. *, *P* < 0.05 in comparison with data collected at 24 h point. **B.** Hematoxylin and eosin morphological staining (H/E) and TUNEL apoptotic cell assay. **C.** Flow cytometry determination of cell cycle distribution and apoptosis in CAOV-3 and OVCAR-3 cells after 120 μM resveratrol treatment for 48 hours.

MTT assay and viable cell counting were performed in 24 hour intervals, which revealed that resveratrol suppressed the growth of CAOV-3 and OVCAR-3 cells in dose- and time-related fashions and CAOV-3 cells were more sensitive to drug treatments (Figure 1A). H/E staining and TUNEL assay showed frequent apoptotic death in resveratrol-treated cell populations (Figure 1B). Flow cytometry demonstrated that 120 μM resveratrol treatment caused distinct G1-phase arrest of the two OC cell lines accompanied with increased apoptotic fractions of OVCAR-3 (16.37%) and CAOV-3 cells (10.11%) at 48 hour time point. As shown in Figure 1C, the G1 and S fractions were 47.0% and 53.0% in normally cultured CAOV-3 cells, which changed to 79.42% and 20.58% in their resveratrol-treated counterpart. Similarly, G1 and S fractions were 40.79% and 59.22% in the normally cultured and 90.34% and 9.66% in the resveratrol-treated OVCAR-3 cells.

### Differential responses of Notch1 and Notch2 to resveratrol

**Figure 2:**
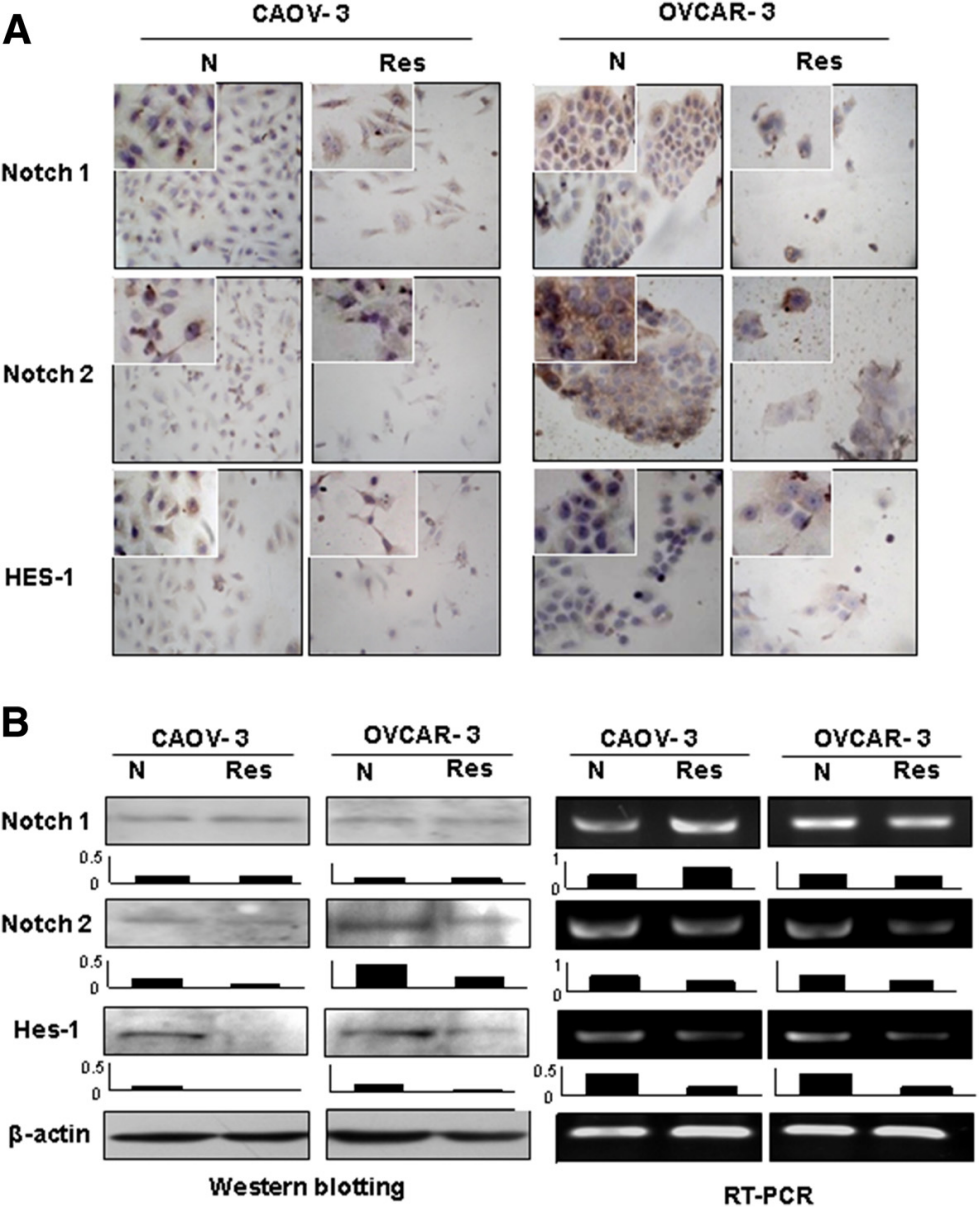
Examination of Notch1, Notch2 and HES1 expression in CAOV-3 and OVCAR-3 cells without (N) and with 48 hour 120 μM resveratrol treatment (Res) by immunocytochemical staining **(A)**, Western blotting and RT-PCR **(B)**. Densitometry analyses were conducted on each of the Western and RT-PCR images.

ICC staining (Figure 2) showed that Notch1 and Notch2 as well as their downstream gene HES1 were expressed in OVCAR-3 and CAOV-3 cells; upon resveratrol treatment, Notch2 and HES1 expression were suppressed in both CAOV-3 and OVCAR-3 cells, while the expression levels of Notch1 was weakly increased in the former and decreased in the later cell line. The results of Western blotting and RT-PCR were in consistence with ICC findings in terms of down-regulated Notch2 and HES1 in both cell lines and the differential Notch1 expression in resveratrol-treated OVCAR-3 and CAOV-3 cells.

### Resveratrol altered β-catenin intracellular distribution patterns

**Figure 3:**
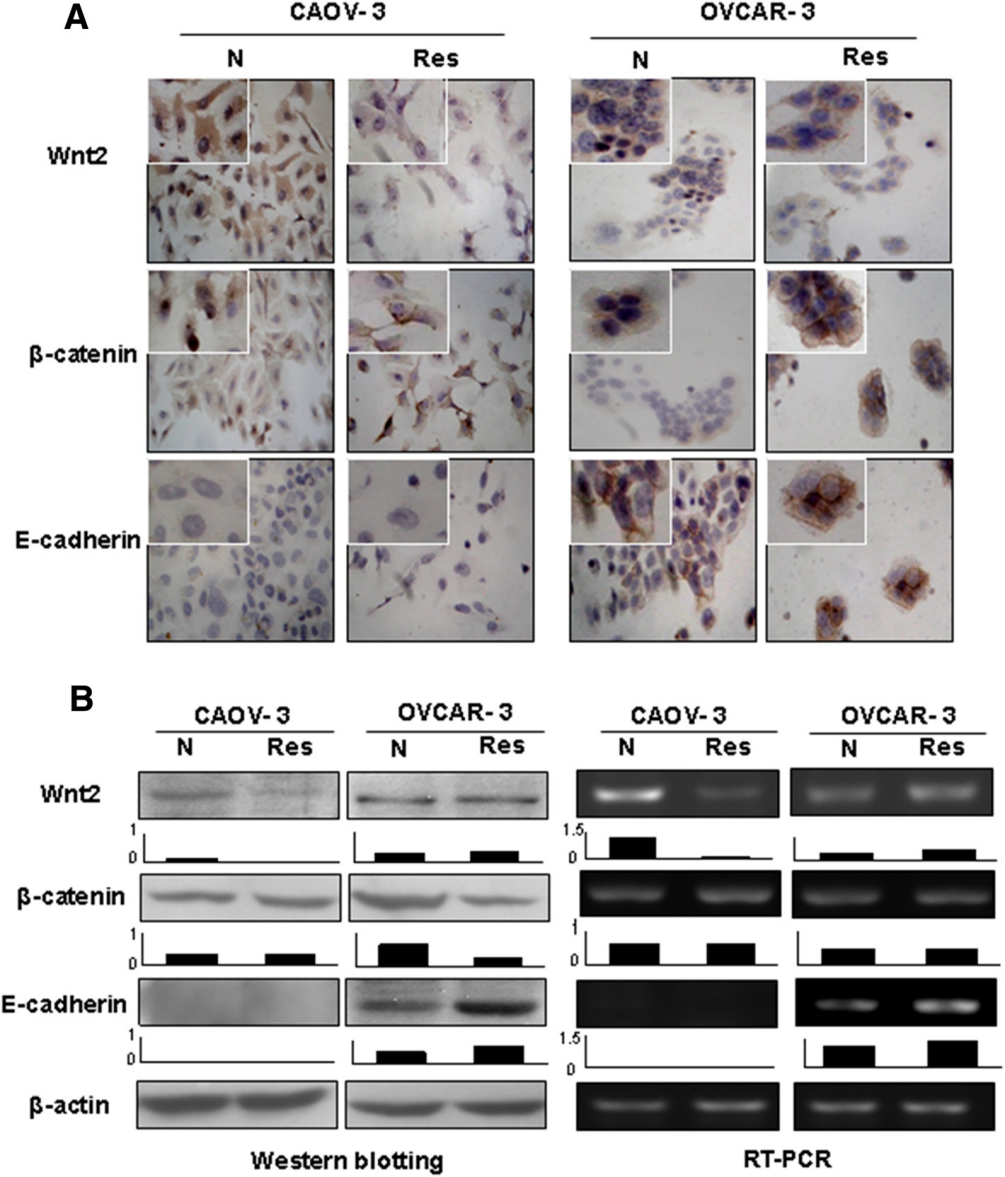
Examination of Wnt2, β-catenin and E-cadherin expression patterns in CAOV-3 and OVCAR-3 cells without (N) and with 48 hour 120 μM resveratrol treatment (Res) by immunocytochemical staining **(A)**, Western blotting and RT-PCR **(B)**. Densitometry analyses were conducted on each of the Western and RT-PCR images.

As shown in Figure 3A, Wnt2 was expressed in OVCAR-3 and CAOV-3 cells. β-catenin proteins were distributed in both the cytoplasmic space and the nuclei of CAOV-3, while they were mainly located in the cytoplasm of OVCAR-3 cells. After resveratrol treatment, Wnt2 was down-regulated (Figure 3B) and nuclear labeling of β-catenin became rare in CAOV-3 cells. In the case of resveratrol-treated OVCAR-3 cells, the level of Wnt2 expression remained almost unchanged, while membrane-labeling of β-catenin was clearly observed. E-cadherin, a β-catenin binding integral protein, was expressed in OVCAR-3 cells and was enhanced after resveratrol treatment; in contrast, it was undetectable in CAOV-3 cells in both RNA and protein levels irrespective to resveratrol treatment.

### Inhibition of STAT3 expression in resveratrol-treated OC cells

**Figure 4:**
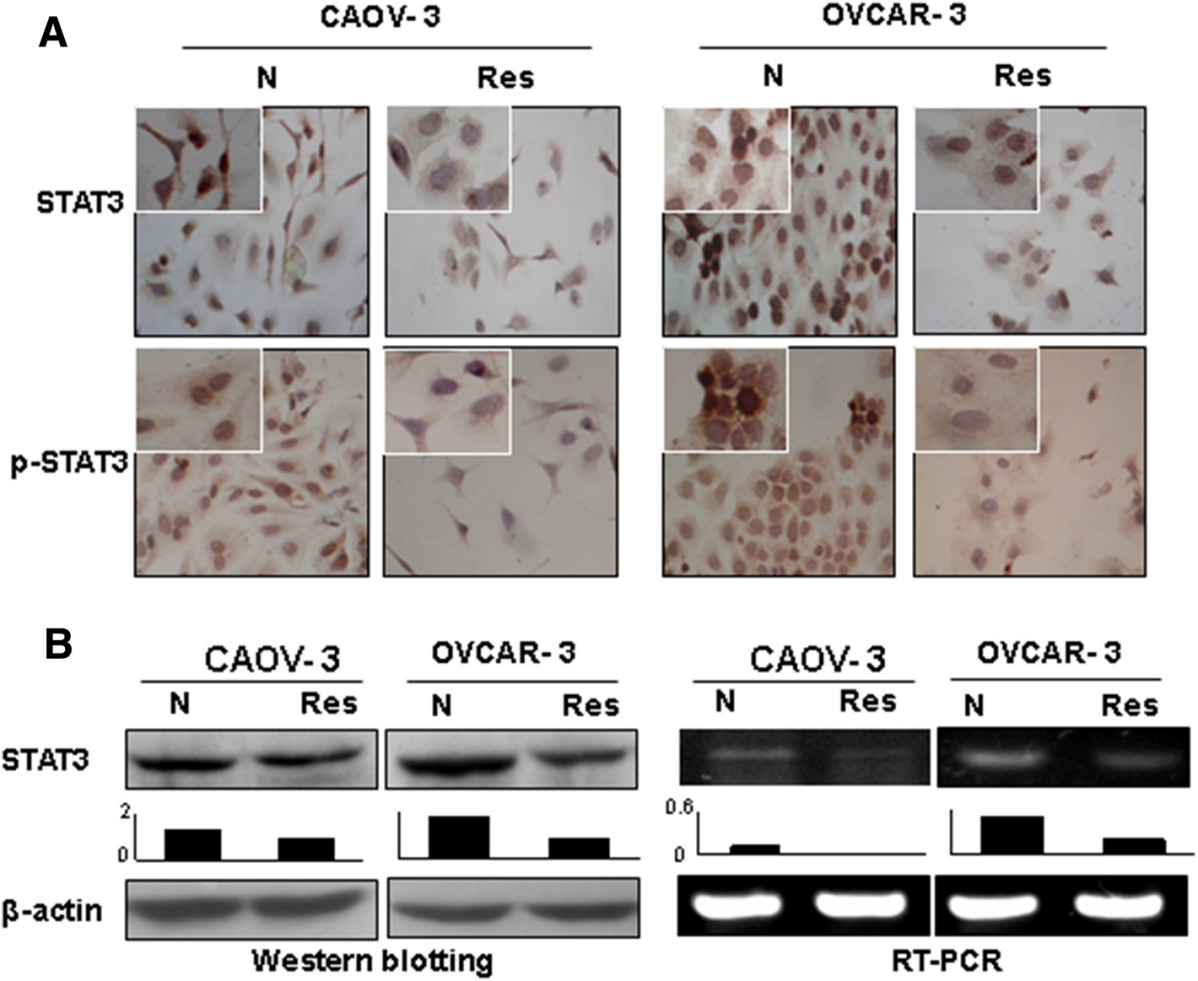
Effects of resveratrol on STAT signaling in ovarian cancer cells. **A.** Immunocytochemical illustration of the levels and intracellular distribution phosphorylated STAT3 in CAOV-3 and OVCAR-3 cells without (N) and with 48 hour 120 μM resveratrol treatment (Res). **B.** Western blot and RT-PCR analyses of STAT3 expression levels performed in parallel with immunocytochemical staining.

The effects of resveratrol on STAT3 signaling in the two OC cell lines were analyzed by immunocytochemical, Western blotting and RT-PCR approaches. It was found that STAT3 was expressed in the normally cultured OVCAR-3 and CAOV-3 cells with distinct nuclear translocation (Figure 4A). STAT3 was down-regulated either in transcriptional or in translational levels in resveratrol-treated ones with reduced nuclear labeling of phosphoralated-STAT3 (Figure 4A and B). For instance, p-STAT3 was predominantly localized in the nuclei of OVCAR-3 cells and became weakened after resveratrol treatment for 48 hours.

### Resveratrol downregulated tumor promoter genes

**Figure 5:**
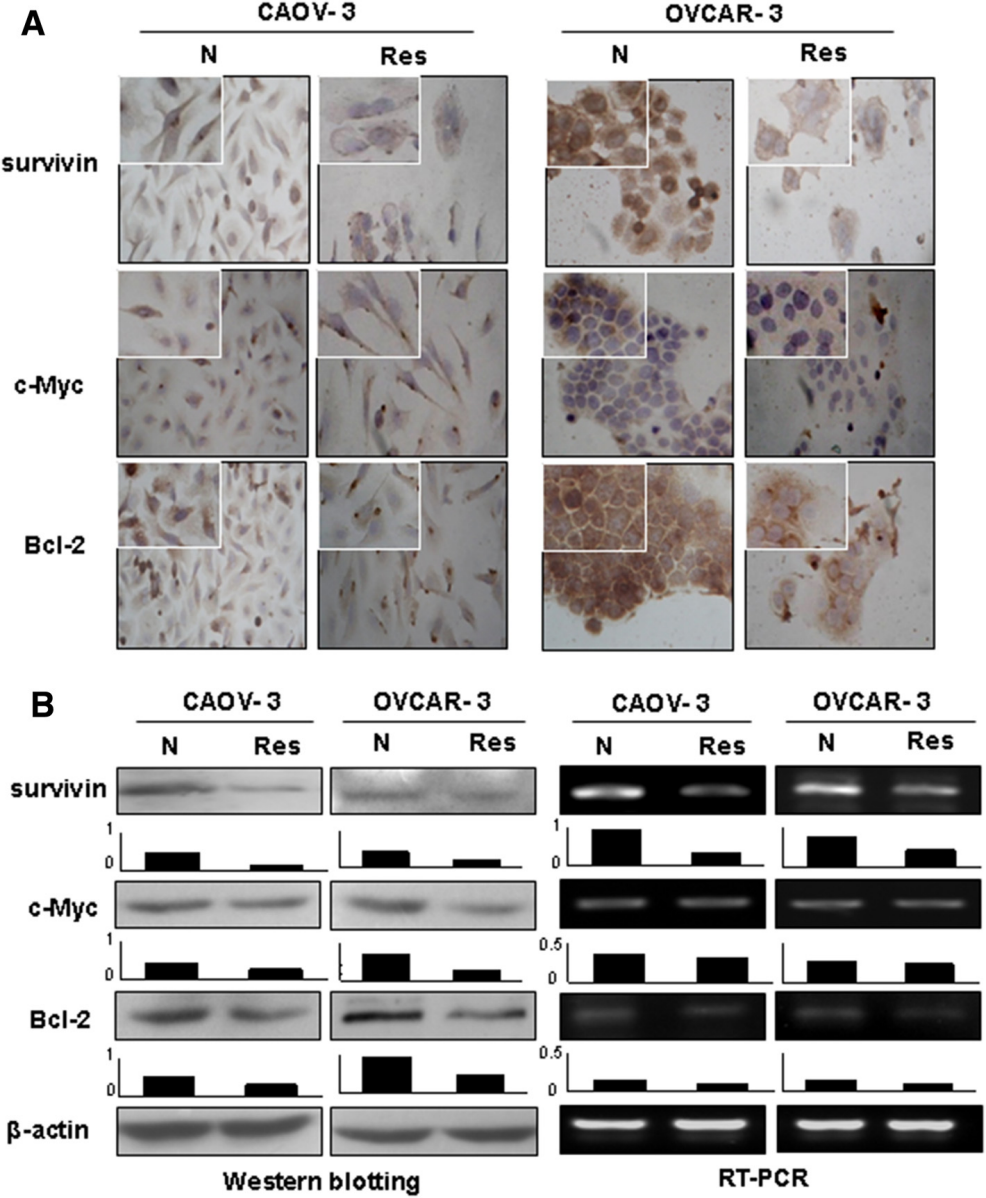
Examination of c-Myc, survivin and Bcl-2 expression in CAOV-3 and OVCAR-3 cells without (N) and with 48 hour 120 μM resveratrol treatment (Res) by immunocytochemical staining **(A)**, Western blotting and RT-PCR **(B)**.

Survivin, c-Myc and Bcl-2 play active roles in cell proliferation and maintenance of ovarian cancers [36-38] and are known as the common target genes of Wnt, Notch and STAT3 signaling [17]. Therefore, their expression statuses in the two OC cell lines without and with resveratrol treatment were analyzed. As shown in Figure 5, the expression levels of c-Myc and especially survivin and Bcl-2 were decreased in both CAOV-3 and OVCAR-3 cells in comparison with their normally cultured counterparts.

### Different effects of selective Notch, Wnt and STAT3 inhibitors

**Figure 6:**
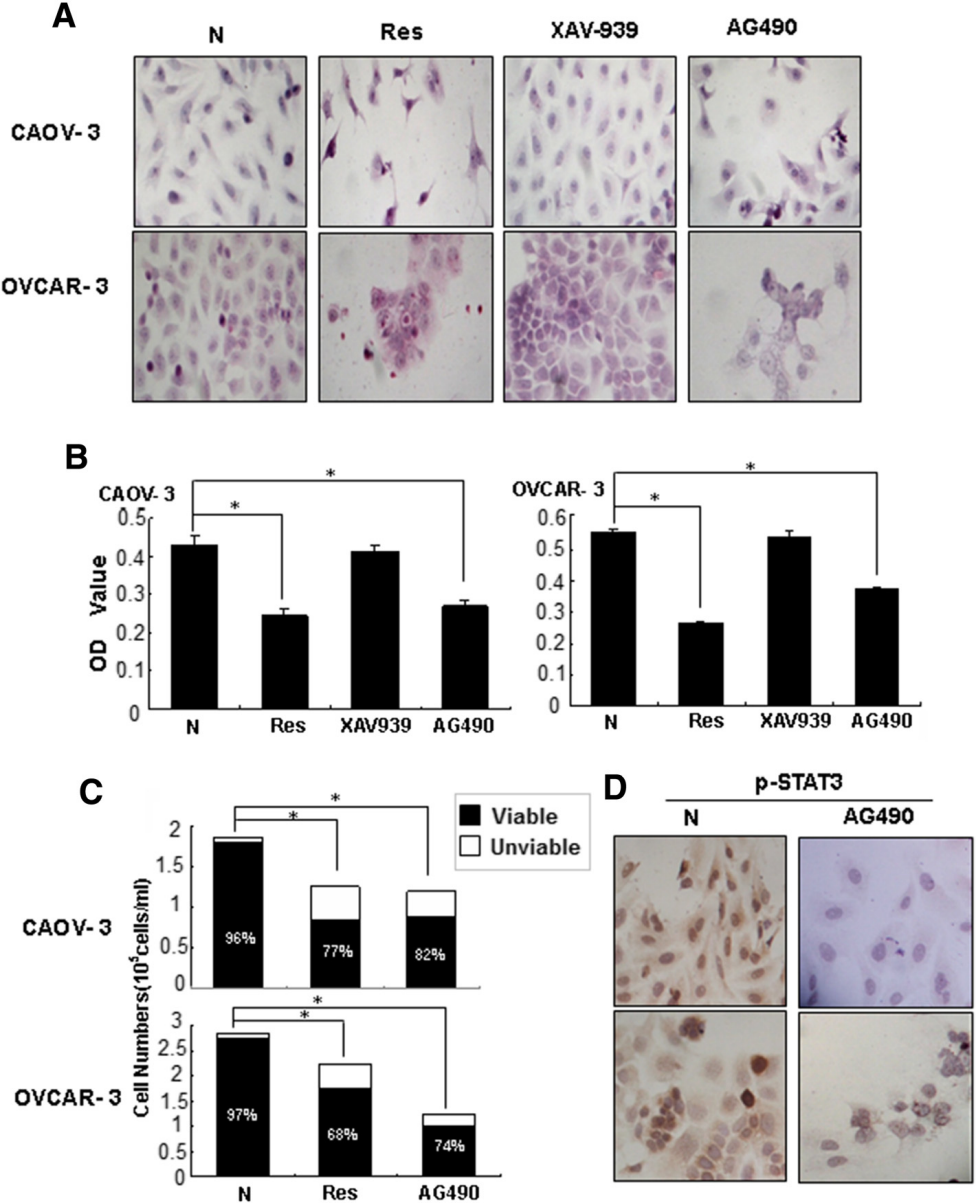
Demonstration of inhibitory effects of selective STAT3 inhibitor AG490 on the proliferation and STAT3 phosphorylation in CAOV-3 and OVCAR-3 cells by Hematoxylin and eosin morphological staining **(A)**, MTT cell proliferation assay **(B)**, viable and unviable cell counting **(C)** and p-STAT3 oriented immunocytochemical staining **(D)**. Normally cultured (N), resveratrol-treated (Res) and Wnt selective inhibitor XAV939-treated cells (XAV939) were cited as normal, effective and ineffective controls, respectively.

To evaluate potential biological significance of activated Notch, Wnt and STAT3 signaling in the two OC cells, the selective inhibitors of the three signaling pathways were used to treat OVCAR-3 and CAOV-3 cells, respectively. The results revealed that although 8 μM L-685,458 blocked Notch activation in the two cell lines in terms of reduced cytoplasmic distribution and nuclear labeling of HES1 proteins, this treatment neither caused growth arrest nor cell death (Data not shown). Wnt inhibitor XAV-939-treated OVCAR-3 and CAOV-3 cells showed reduction of cytoplasmic distribution and nuclear translocation of β-catenin but no distinct morphologic change and growth inhibition could be observed in comparison with their normally cultured counterparts (Figure 6A and B). STAT3 phosphorylation was inhibited in OVCAR-3 and CAOV-3 cells upon 80 μM AG490 treatment for 48 hours, accompanied with similar growth suppression rates (74%) as that of resveratrol-treated populations (68%) in OVCAR-3 cells and 82% versus 77% in CAOV-3 cells (Figure 6A-C).

## Discussion

Ovarian cancer is one of the most lethal malignancies due to its strong spreading tendency via different dissemination routes including peritoneal implantation [6,39]. Because of the difficulty to remove cancer cells radically, adjuvant chemotherapy is employed to reduce the risk of tumor relapse [40]. Nevertheless, the therapeutic outcome of OC patients is not optimistic due to the frequent drug resistance of cancer cells and severe toxic effects of anticancer drugs [41,42]. Apparently, more effort should be made to explore safer and more effective agent for ovarian cancer patients. It has been known that the anticancer doses of resveratrol are non-toxic to some kinds of normal cells/tissues [15,43], suggesting the potential values of this compound in the treatment of human cancers. Resveratrol also exerts inhibitory effects on ovarian cancer cells [21,22]. The current study further demonstrates that the resveratrol sensitivities of ovarian cancer cells are not identical, because 100 μM resveratrol is sufficient to cause G1 phase arrest and remarkable apoptosis in CAOV-3 while a dose of 120 μM is required to induce similar cellular events in OVCAR-3 cells. Although the underlying reason(s) leading to the differential resveratrol sensitivities remains to be disclosed, our results have potential translational values because 1) both OVCAR-3 and CAOV-3 cells are resistant to cis-platinum (cis-diamminedichloroplatinum), a commonly used agent in anti-OC therapy [43,44], 2) 120 μM resveratrol is harmless to normal neural and urothelial cells in vivo [15,45] and 3) long-term intra peritoneal administration of 150 mg (657 μM)/kg/per day resveratrol dose not affect the life quality of the rats including the reproductive ability [Li-Xia Zhong et al. unpublished data]. In this context, it would be considered that resveratrol may be a promising candidate to treated ovarian cancers, especially those with cis-platinum resistance.

A body of evidence suggests that many molecular alterations occur during ovarian carcinogenesis, of which the activated Notch, Wnt and STAT3 signaling pathways are supposed to play active roles in the carcinogenic process by up-regulating the expression of some tumor promoting genes such as c-Myc, survivin and Bcl-2 [46,47]. On the other hand, resveratrol possesses multifaceted targeting capacities [48,49] and the cancer-associated signaling pathways mediated by STAT3, Wnt2 and/or Notch1/2 have been known as its molecular targets [35]. Although the inhibitory effects of resveratrol on ovarian cancer cells have been documented, the critical molecular event(s) caused by resveratrol remain largely unknown. In this study, the statuses of STAT3, Notch and Wnt2 signaling in OVCAR-3 and CAOV-3 cells and the influences of resveratrol in them are investigated. It is revealed that all of the three signaling pathways are activated in the two cell lines because of the co-existence of nuclear translocations of phosphorylated STAT3 (p-STAT3), HES1 and β-catenin. After resveratrol treatment, nuclear labeling of p-STAT3, β-catenin and HES1 become rare and weakened, indicating the concurrent inhibition of the biological activities of STAT3, Notch and Wnt2 signaling by this multi-targeting compound and its correlation with the suppressed growth of ovarian cancer cells.

c-Myc, Bcl-2 and survivin are well known cancer promoter genes and their transcription can be triggered by STAT3, Notch and Wnt signaling, respectively [18,46,47]. In accompany with distinct growth arrest and apoptosis, the expression of these three genes is down-regulated in resveratrol-treated OVCAR-3 and CAOV-3 cells, which may be considered as the consequence of concurrent STAT3, Notch and Wnt inactivation. It has been recognized that reduction or absence of membrane E-cadherin distribution indicates the dedifferentiation states of epithelial cells and is one of the major reasons of β-catenin cytoplasmic accumulation and nuclear translocation [50,51]. In resveratrol treated OVCAR-3 cell population, the level of E-cadherin expression is increased and more abundant β-catenin membrane distribution can be observed, indicating the favorable effects of resveratrol on cell differentiation and inhibitory effect on Wnt signaling. Based on this notion, the lack of membranous β**-**catenin labeling of resveratrol-treated CAOV-3 cells would be largely due to the silenced E-cadherin expression. Even though, CAOV-3 cells remain sensitive to resveratrol, suggesting that the presence or absence of E-cadherin expression and β**-**catenin nuclear translocation may not be the critical factor in determining the responsiveness of OC cells to resveratrol.

Although resveratrol suppressed STAT3, Notch and Wnt activations have been evidenced in OVCAR-3 and CAOV-3 cells, it is still unclear which of them are/is closely linked with resveratrol-caused cell crisis. To address this issue, the two ovarian cancer cells are treated by selective inhibitors of Notch, Wnt and STAT3 respectively and their responses are compared with that of their resveratrol-treated and normally cultured counterparts. The results reveal that Notch inhibitor L-685,458 and Wnt inhibitor XAV-939 efficiently block nuclear translocations of HES1 and β-catenin, but neither of them leads to distinct growth inhibition of OVCAR-3 and CAOV-3 cells. On the other hand, STAT3 inhibitor AG490 suppresses proliferation and induces apoptosis in the extents as similar as that of resveratrol-treated cells. These findings thus suggest 1) the critical roles of STAT3 activation in the growth and survival of human ovarian cancer cells and 2) STAT3 signaling as the common oncotarget of resveratrol in cancer cells with different origins [52]. Although simply block of Notch or Wnt signaling transduction has little inhibitory effect on the two OC cell lines so far checked, the implications of their inactivation can not been overlooked because of their compensatory roles in regulating the expression of ovarian cancer-related genes including the ones examined in this study.

## Conclusion

Our current study demonstrate the efficiencies of resveratrol in inhibiting human ovarian cancer cells in terms of remarkable G1 phase accumulation, increased apoptosis fraction and concurrent suppression of Wnt, Notch and STAT3 signaling as well as their downstream cancer-related gene expression. Treatments with Wnt, Notch or STAT3 selective inhibitor reveal that only AG490, a JAK-specific inhibitor, inhibits OVCAR-3 and CAOV-3 cells in the extent as similar as that of resveratrol, suggesting the importance of STAT3 activation in the maintenance and survival of ovarian cancer cells. It is therefore possible that the activated STAT3 signaling is the critical molecular target of resveratrol and this polyphenol compound would be an alternative option in the management of ovarian cancers, especially the ones insensitive to conventional therapeutic drugs.
